# Homocysteine-induced decrease in HUVEC cells’ resistance to oxidative stress is mediated by Akt-dependent changes in iron metabolism

**DOI:** 10.1007/s00394-020-02360-8

**Published:** 2020-08-13

**Authors:** Andzelika Borkowska, Wieslaw Ziolkowski, Katarzyna Kaczor, Anna Herman-Antosiewicz, Narcyz Knap, Agata Wronska, Jedrzej Antosiewicz

**Affiliations:** 1 Department of Bioenergetics and Physiology of Exercise, Medical University of Gdansk, 1 Debinki St., 80-210 Gdańsk, Poland; 2 Department of Rehabilitation Medicine, Medical University of Gdansk, 80-219 Gdańsk, Poland; 3 Department of Medical Biology and Genetics, Faculty of Biology, University of Gdansk, 80-308 Gdańsk, Poland; 4 Department of Medical Chemistry, Medical University of Gdansk, 80-210 Gdańsk, Poland; 5 Department of Histology, Medical University of Gdansk, 80-210 Gdańsk, Poland

**Keywords:** Ferritin, Insulin signaling, Stress response

## Abstract

**Purpose:**

Hyperhomocysteinemia is an independent risk factor for cardiovascular diseases and also promotes neuronal death in various neurodegenerative diseases. There is evidence that iron can mediate homocysteine (Hcy) toxicity. Thus, the aim of this study was to investigate the effect of Hcy on iron metabolism in HUVEC and SH-SY5Y cells.

**Methods:**

HUVEC and SH-SY5Y cells were treated with 3 mM Hcy for a defined time.

**Results:**

We demonstrate that Hcy induced the upregulation of ferritins type L and H in HUVEC cells in a time-dependent manner and had no effect on the ferritins in SH-SY5Y cells. The change in ferritin expression was preceded by a significant decrease in the cellular level of the active form of Akt kinase in HUVEC but not in SH-SY5Y cells. An increase in ferritin L and H protein levels was observed in the Akt1, Akt2, Akt3 siRNA transfected cells, while in the cells transfected with FOXO3a siRNA, a decrease in both ferritins levels was noticed. Moreover, in the HUVEC cells treated with Hcy for 6 days, the active form of kinase Akt returned to the control level and it was accompanied by a drop in ferritin L and H protein levels. Cytotoxicity of hydrogen peroxide significantly increased in HUVEC cells pre-treated with Hcy for 24 h.

**Conclusions:**

These data indicate that Hcy induces an increase in cellular ferritin level, and the process is mediated by alterations in Akt-FOXO3a signaling pathway.

## Introduction

Hyperhomocysteinemia is an independent risk factor for cardiovascular diseases, such as ischemic heart disease, stroke, peripheral vascular disease or atherosclerosis [1]. Moreover, according to recent reports, an elevated Hcy level in plasma is related to the development of various neurodegenerative diseases, including Alzheimer's disease, dementia, mild cognitive disorders and others [2] [3]. However, the molecular mechanism of homocysteine (Hcy) action has not been well understood. It was demonstrated that Hcy affected multiple signaling pathways, including extracellular signal-regulated kinase (ERK), Dyrk1A serine/threonine kinase involved in diverse cellular processes, PI3K/Akt pathway, stress-activated protein kinase, and others. For example, hyperhomocysteinemia in rats was shown to be associated with the inactivation of PI3K/Akt signaling pathway, and the deleterious effects of Hcy were reversed by Akt kinase activators [4–6]. These data suggest that Akt inactivation plays an essential role in homocysteine toxicity. One of the Akt substrates is eNOS whose phosphorylation at Ser1177 leads to the activation of the enzyme, and consequently to an increased NO synthesis in the cell [7]. Analogously, studies on humans and animals demonstrated that insulin resistance which is a marker of the metabolic syndrome leads to a decrease in Akt activity [8]. Interestingly, both hyperhomocysteinemia and insulin resistance were shown to be accompanied by changes in iron metabolism. Importantly, iron sucrose worsens and iron chelation alleviates Hcy-dependent decrease in flow-mediated vasodilation [9]. An excessive tissue iron accumulation, among others in arterial walls, is associated with an increased risk of some morbidities, like the coronary artery heart disease, cancer, diabetes mellitus and others [10]. High iron storage in the body was proven to be a risk factor for insulin resistance and metabolic syndrome [11]. However, the involved molecular mechanisms have not been clarified. Iron is safely stored in a cell by ferritin, and the cell can adapt to an increased iron level by enhancing ferritin biosynthesis [12]. Conversely, ferritin iron can contribute to the labile iron pool (LIP). Recently, we have demonstrated that under the conditions of oxidative stress ferritin undergoes proteasomal degradation and LIP expansion. Moreover, we demonstrated that the process is regulated by oxidative stress-activated protein kinases and an adaptor protein p66Shc [13, 14]. In light of these reports, it is clear that the amount of stored iron may determine Hcy toxicity as has been previously suggested [9]. Therefore, understanding the effect of Hcy on iron metabolism is crucial to elucidate the cellular mechanism of Hcy toxicity. Considering that the main targets of Hcy toxicity are arteries and neurons we used two cell lines HUVEC and SH-SY5Y being endothelial and neuronal ones, respectively. The purpose of the study was to determine whether Hcy may influence HUVEC and SH-SY5Y ferritin protein level, and whether Akt kinases play a role in the process. The inactivation of Akt kinase has been reported to lead to the activation of FOXO3a transcriptional factor which in turn upregulates antioxidant proteins. It has been shown that DAF-16, which is an invertebrate homolog of Forkhead family of FOXO transcriptional regulators can upregulate ferritin H in *Caenorhabditis elegnas* [15]. Therfore, we hypothesized that Hcy may induce changes in iron metabolism and the cell resistance to oxidative stress by modulation of Akt/FOXO3a/ferritin signaling pathway.

## Materials and methods

### Reagents

Endothelial Cell Growth Medium (211-500) was purchased from the European Collection of Authenticated Cell Cultures (ECACC, Porton Down, UK). DMEM/F12 (Sigma 51445C), antibiotic mixture, fetal bovine serum, trypsin–EDTA were purchased from Sigma-Aldrich Ltd (Poznan, Poland). The antibodies against ferritin H (sc-25617) and L (sc-74513), p-Akt (Ser-473) (sc-7985-R), Akt (sc-8312), siRNA Akt 1, 2, 3 (sc-2925; sc-29197; sc-38912), control (sc-37007) and all the reagents for siRNA transfection were obtained from Santa Cruz Biotechnology (Dallas, USA). Antibodies against FOXO3a (ab53287) and p-FOXO3a (Ser 253) (ab154786) were from Abcam (Cambridge, UK). Homocysteine (H4628), insulin (I9278), deferoxamine (DFO) (D9533), trypan blue solution (T8154), secondary antibodies anti-Rabbit IgG—Peroxidase (A9169) and anti-Mouse IgG—Peroxidase (A9044) as well as antibodies against β-actin (A3854) were from Sigma-Aldrich Ltd (Poznan, Poland).

### Cell culture

HUVEC (Human Umbilical Vein Endothelial Cells) (200-05 N) were purchased from European Collection of Authenticated Cell Cultures (ECACC, Porton Down, UK). SH-SY5Y (Human bone marrow cells) (ATCC^®^ CRL-2266™) were purchased from American Type Culture Collection (Manassas, VA, USA). The cells were maintained at 37 °C in an atmosphere of 95% air and 5% CO_2_. For treatment procedure cells were seeded on a plate in an adequate amount and allowed to attach overnight. On the next day the cells were treated with 3 mM Hcy, 200 nM insulin, 25 µM DFO and 300 µM hydrogen peroxide in various combinations.

### siRNA transfection

The cells were seeded at a density of 1.5 × 10^6^ per 6-cm plate and allowed to attach overnight. At 50–60% confluence the mix of each siRNA duplex (siRNA Control, Akt1, Akt2, Akt3 and FOXO3a) and siRNA Transfection Reagent (sc-295828, Santa Cruz Biotechnology, Dallas, USA) were administered. 6 h after transfection, the cells were supplied with the medium containing serum and antibiotics at concentrations 2 times higher than standard. On the following day, (20 h after medium addition) media were replaced with the fresh ones. 48 h after transfection, cells were harvested for assays.

### Gene expression

Total cellular RNA was isolated using the Total RNA mini Plus Concentrator (036-25C, A&A Biotechnology, Gdynia, Poland). 1 µg of total RNA was reverse-transcribed with the use of RevertAid Reverse Transcriptase (EP0441, Thermo Fischer Scientific, Fitchburg, WI, USA) and 0.5 µg oligo(dT)_18_ primers (Sigma-Aldrich, Munich, Germany). Real-time quantitative PCR (qPCR) method was applied to measure the gene expression of ferritin L (*FTL*) and ferritin H (*FTH1*), normalized to Ribosomal Protein L32 (*RPL32*) gene as an internal control. The cDNA matrices were amplified in StepOne Plus thermal cycler (Life Technologies-Applied Biosystems, Grand Island, NY, USA) using SensiFastSybr™ No-Rox kit (BIO-98005, Bioline, London, UK) containing 0.2 µM sense and antisense primers. The primers used were:

#### *FTL*: R:5′TCCTACGTTTACCTGTCCATGT3′, F:5′GTTTGTGCAGTTCCAGTAGTGA3′;

#### *FTH*: R:5′TCCTACGTTTACCTGTCCATGT3′, F:5′GCCAATTCGCGGAAGAAGTC3′;

#### *RPL32: *R:5′GCGCCACCGTCCCTTCTCTC3′, F:5′GACATATCGGTCTGACTGGTGC3’.

Relative quantities of transcripts were calculated with the ${2}^{{ - \Delta \Delta C_{{\text{T}}} }}$ formula according to Livak’s metod [16].

### Immunoblotting

HUVEC cells were treated as described above. For 6 and 2 day incubation with Hcy, HUVEC cells were normally cultured in the medium with or without homocysteine. The two-day incubation was started on day 4 of the 6-day incubation. Both floating and attached cells were collected on the same day, washed in PBS, resuspended in a lysis solution containing 50 mmol/L Tris–HCl (pH 7.5), 150 mmol/L NaCl, 1% (v/v) Triton X-100, 0.1% (w/v) SDS, and incubated for 40 min on ice with gentle shaking. The cell lysate was cleared by centrifugation at 16 000 g for 20 min. Lysate proteins were resolved by 10–12% SDS-PAGE. The proteins were then transferred onto a membrane by a standard semi-dry technique. After blocking the membrane with 5% (w/v) solution of dried skim milk dissolved in TBS buffer containing 0.1% (v/v) Tween 20, it was incubated with a desired primary antibody overnight at 4 °C. The membrane was then treated with an appropriate secondary antibody, and the immunoreactive bands were visualized using the enhanced chemiluminescence method. Changes in protein level were assessed by densitometric scanning of the bands and corrected for β-actin loading control.

### Cell viability

For 6-day and 24 h incubation with Hcy, HUVEC cells were normally cultured and passaged in medium with or without homocysteine. The 24 h incubation with Hcy was started on day 5 of the 6-day incubation and all of the cells were collected on the same day. After incubation with Hcy the cell medium was replaced with the new one containing or not containing 300 μM hydrogen peroxide, and the incubation continued for 24 h. Then the cells were harvested and stained with the DNA-binding dye trypan blue for 5 min. Trypan blue-positive cells were counted using the automated cell counter Luna (Logos Biosystems).

### Statistical analysis

Statistical analyses were performed using Graphpad Prism. Data were presented as mean ± SEM. The differences between the means were determined by *t* Student test or one-way analysis of variance (ANOVA). The post hoc Tukey or Dunnett multiple comparison tests were performed to identify significantly different groups. The significance level *p* < 0.05 was accepted for analysis. All of the presented data were collected from at least three independent experiments.

## Results

### Homocysteine decreases Akt phosphorylation in HUVEC cells

**Fig. 1:**
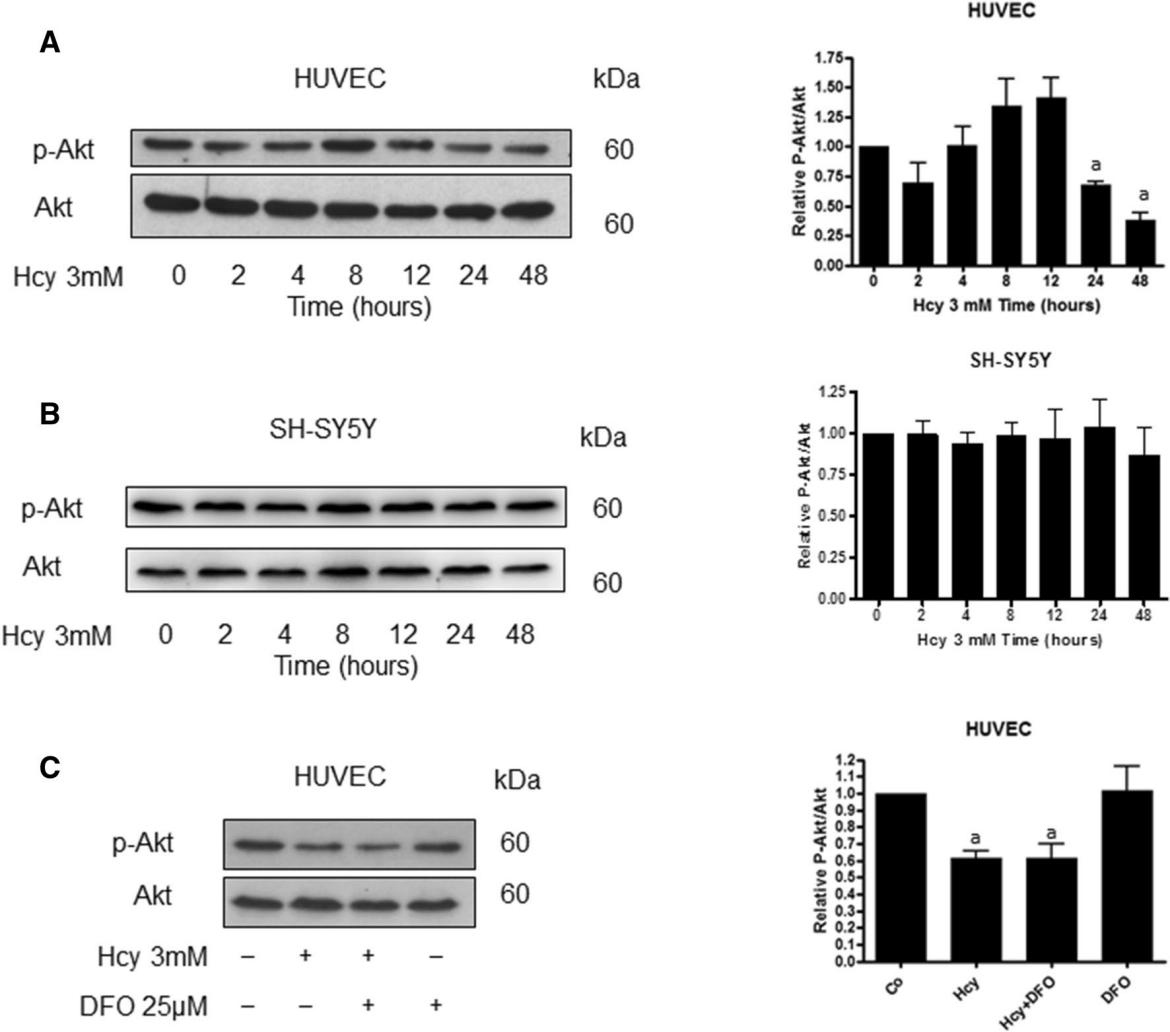
Different effect of homocysteine on Akt activity in HUVEC and SH-SY5Y cell lines. Immunoblotting for Akt and p-Akt using lysates from **A** HUVEC, **B** SH-SY5Y cells having been treated with 3 mM Hcy for the indicated time periods. Blots were stripped and reprobed with anti-β-actin antibody to normalize for differences in protein loading. Bar graphs on the right panel of the figure present the mean values ± SEM of three independent experiments relative to control where “a”, represents significant difference (*p* < 0.05) relative to control (one-way ANOVA followed by Dunnett’s multiple comparison test). **C** Immunoblotting for Akt and p-Akt using lysate from HUVEC cells following treatment of HUVEC cells for 24 h with 3 mM Hcy, with or without a 2 h pre-treatment with 25 μM DFO. Blots were stripped and reprobed with anti-β-actin antibody to normalize for differences in protein loading. Significance of differences between samples was evaluated by one-way ANOVA followed by Tukey’s multiple comparison test. Bar graphs on the right panel of the figure present the mean ± SEM of three independent experiments relative to control where “a” represents statistical significance of *p* < 0.05 as compared to control

Hcy treatment caused a decrease in Akt phosphorylation at Ser-473 which is crucial for Akt activation in HUVEC cells at relatively early time points. The p-Akt increased slightly above the control level at 4 through 12 h, and then significantly decreased at 24 and 48 h of treatment (Fig. 1A). On the other hand, Hcy had no effect on Akt phosphorylation in SH-SY5Y cell line (Fig. 1B). To evaluate the role of iron in Hcy-induced Akt inactivation, the cells were pre-treated with a specific iron chelator, deferoxamine (DFO). DFO-treated cells demonstrated lower levels of Akt and p-Akt with no effect on Hcy-induced drop in Akt phosphorylation (Fig. 1C).

### Hcy-induced upregulation of ferritin L and H is mediated by Akt protein kinase

**Fig. 2:**
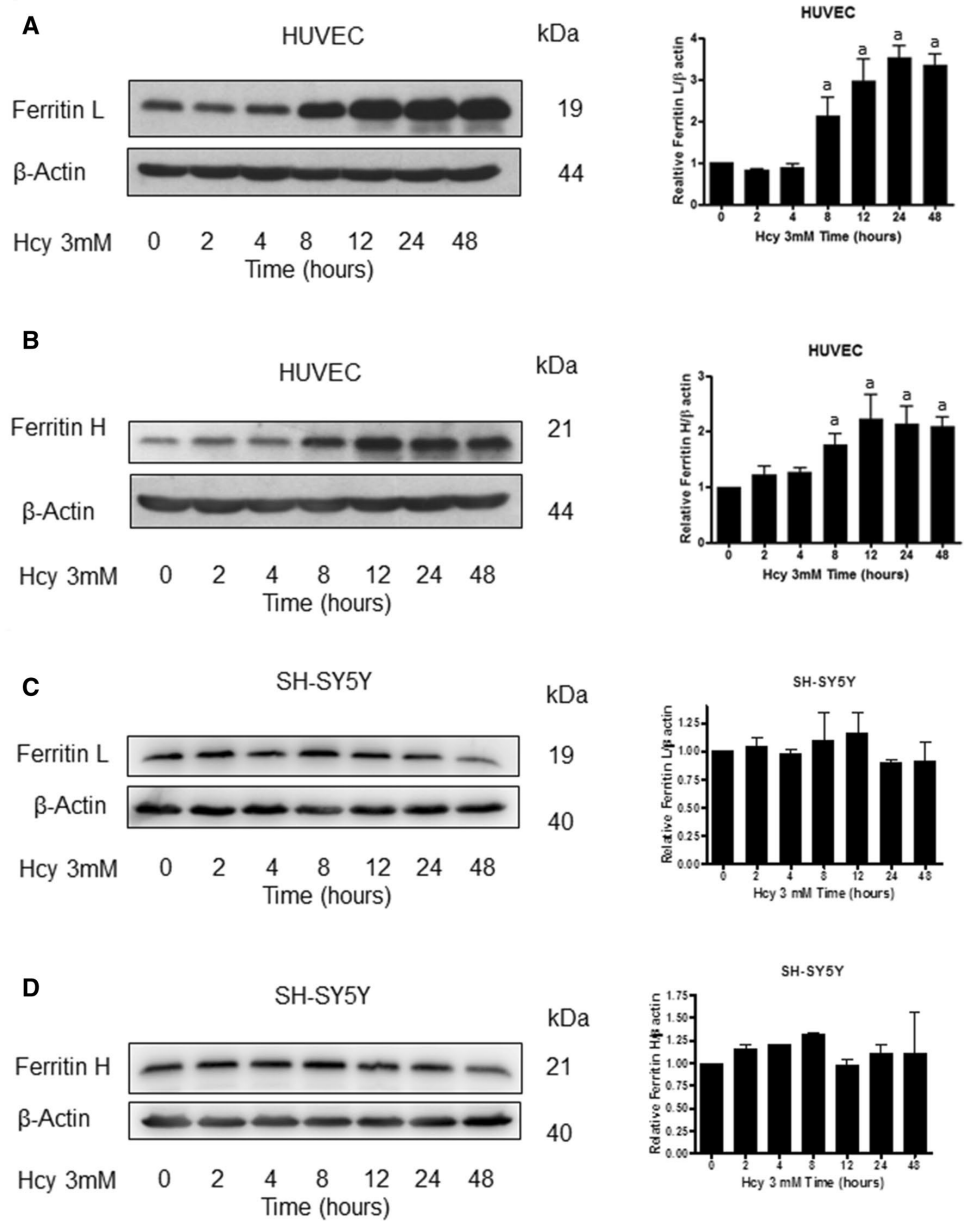
Homocysteine-induced increase in ferritin L and H levels in HUVEC cells. Immunoblotting for **A** ferritin L and **B** ferritin H using lysate from HUVEC cells having been treated with 3 mM Hcy for the indicated time periods. Immunoblotting for **C** ferritin L and **D** ferritin H using lysate from SH-SY5Y cells having been treated with 3 mM Hcy for the indicated time periods. Blots were stripped and reprobed with anti-β-actin antibody to normalize for differences in protein loading. Graphs on the right panel of the figure present the mean ± SEM of three independent experiments relative to control where "a" represents statistical significance at *p* < 0.05 as compared to control (one-way ANOVA followed by Dunnett’s multiple comparison test)

Hcy induced a slight decrease in ferritin L level (Fig. 2A) and an increase in ferritin H level (Fig. 2B) as early as after 2 h of treatment. However, starting from 8 h of treatment onward a significant rise in both, ferritin L and H levels was observed. Conversely, Hcy did not induce changes in ferritin protein levels in SH-SY5Y cell line (Fig. 2C, D). Simultaneously, a decrease in p-Akt level (representing the active form of the enzyme) was observed in HUVEC but not in SH-SY5Y cells (Fig. 1A, B). Our next goal was to establish whether Akt is involved in the regulation of ferritin protein level.

**Fig. 3:**
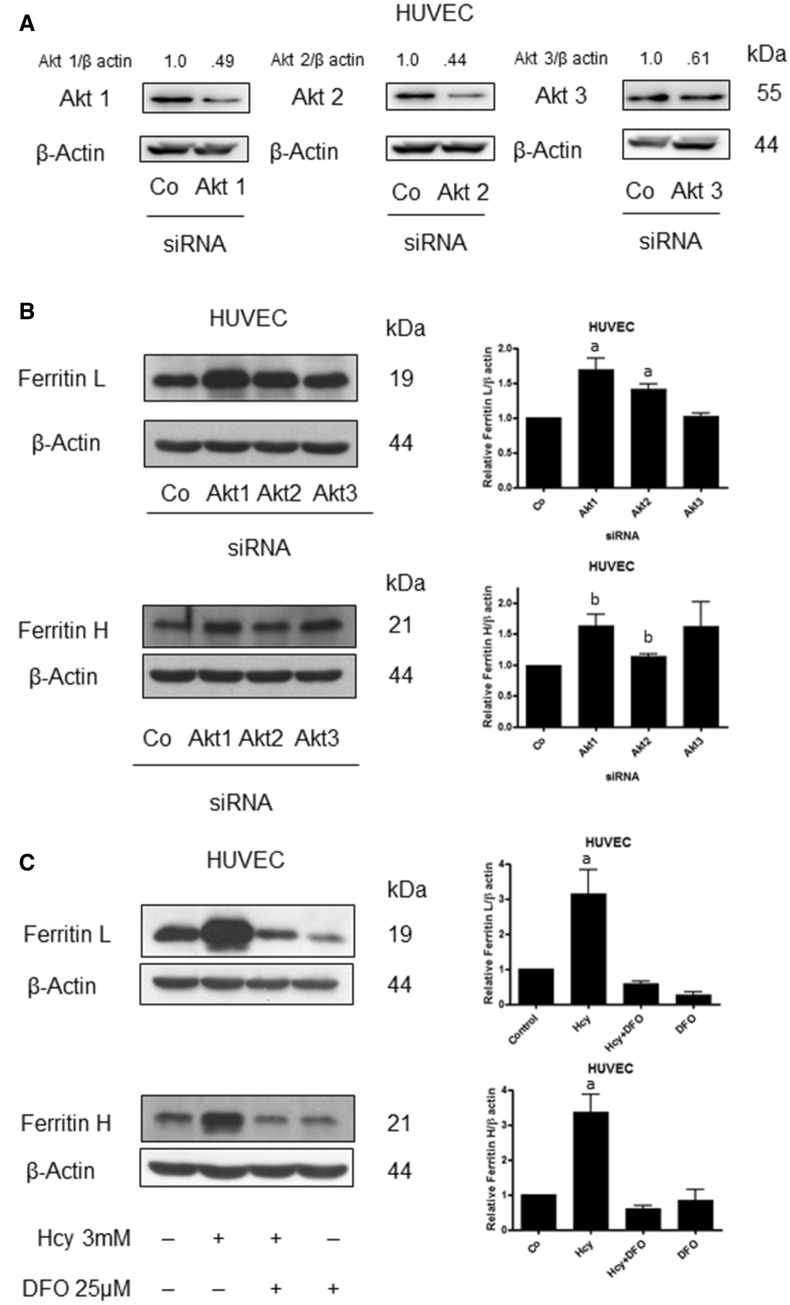
Downregulation of Akt leads to a rise in ferritin L and H in HUVEC cells. **A** Immunoblotting for Akt1, Akt2, Akt3 using lysate of HUVEC cells transfected with siRNA against Akt1, Akt2 and Akt3, respectively. Densitometric scanning data after correction for actin loading control are on top of Akt immunoreactive bands. **B** Immunoblotting for ferritin L and ferritin H using lysate of HUVEC cells transfected with siRNA against Akt1, Akt2 and Akt3, respectively, Blots were stripped and reprobed with anti-β-actin antibody to normalize for differences in protein loading. Bar graphs on the right panel of the figure present the mean ± SEM of three independent experiments relative to control where: "a" represents statistical significance at *p* < 0.05 as compared to control (one-way ANOVA followed by Dunnett’s multiple comparison test), "b" represents statistical significance at *p* < 0.05 as compared to control (*t* Student test). **C** Immunoblotting for ferritin L and ferritin H using lysate of HUVEC cells treated for 24 h with 3 mM Hcy with or without a 2 h pretreatment with 25 μM DFO. Blots were stripped and reprobed with anti-β-actin antibody to normalize for differences in protein loading. Bar graphs on the right panel of the figure present the mean ± SEM of three independent experiments relative to control where "a: represents statistical significance at *p* < 0.05 as compared to control (one-way ANOVA followed by Tukey’s multiple comparison test)

HUVEC cells were transfected with a specific siRNA to silence Akt1, Akt2 or Akt3 (Fig. 3A). Silencing of Akt1 and Akt2 was accompanied by a rise in ferritin L level while downregulation of Akt3 had no effect on ferritin L protein level (Fig. 3B). In the case of ferritin H, the increase in its level was observed in the cells with downregulated Akt1, Akt2 or Akt3 (Fig. 3B). These data indicate that a decrease in the active Akt as observed in Hcy-treated HUVEC cells is associated with ferritins upregulation. In the cells treated with DFO a significant drop in ferritin L and ferritin H levels was observed, and co-treatment with Hcy had a distinctive effect on the ferritins level. The level of ferritin L was increased by Hcy in DFO pre-treated cells compared to DFO only treated cells, while in the case of ferritin H its level was not affected (Fig. 3C).

### Insulin reversed Hcy-induced changes in ferritin protein level

**Fig. 4:**
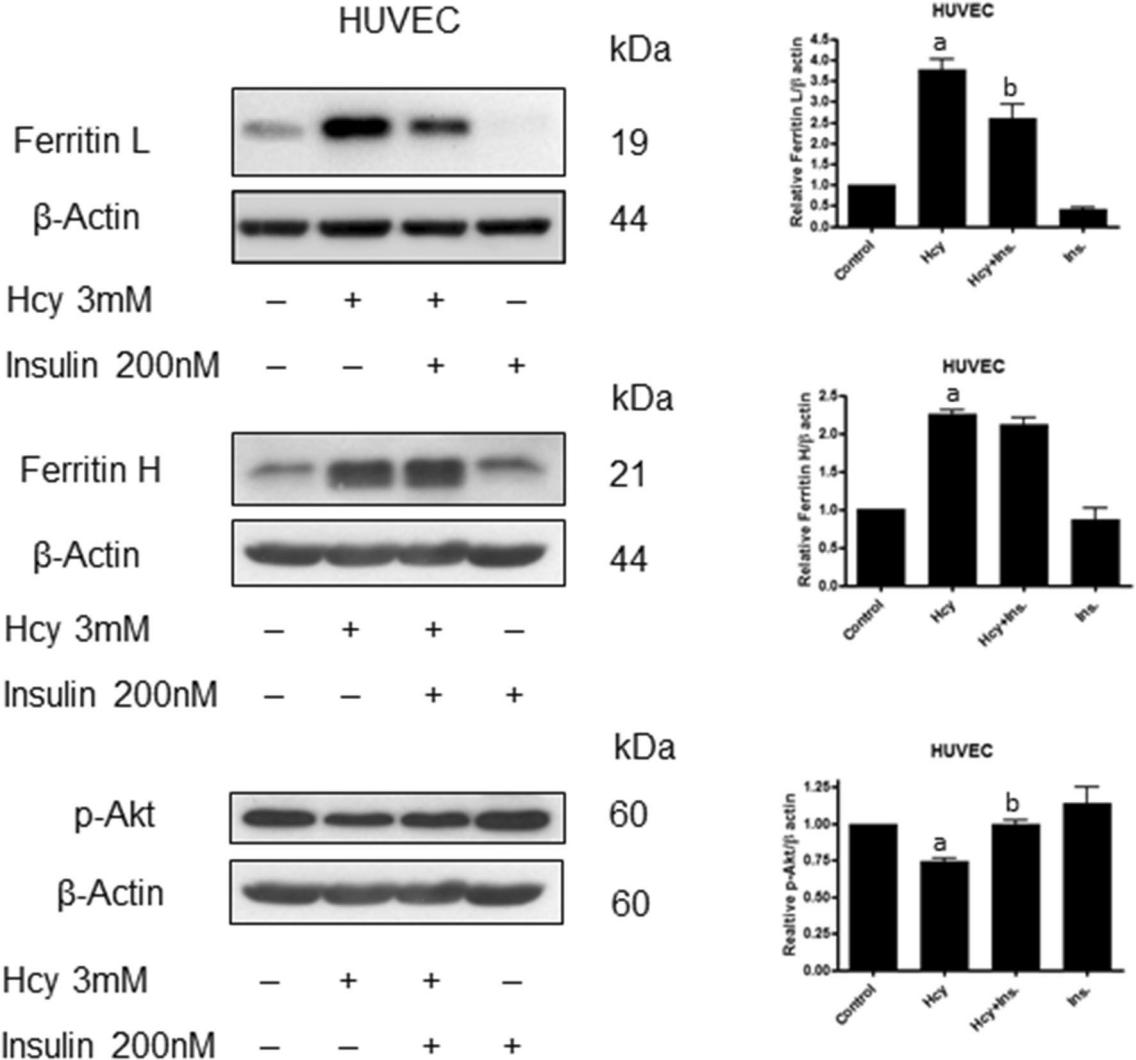
Effects of insulin on homocysteine-induced ferritin L and H. Immunoblotting for ferritin L, ferritin H and p-Akt using lysate from HUVEC cells treated for 24 h with 3 mM Hcy, and with or without 200 nM insulin. Blots were stripped and reprobed with anti-β-actin antibody to normalize for differences in protein loading. Bar graphs on the right panel of the figure present the mean ± SEM of three independent experiments relative to control where "a" represents statistical significance at *p* < 0.05 as compared to control (one-way ANOVA followed by Tukey’s multiple comparison test)

To confirm the role of Akt kinase in the regulation of ferritin protein level, the effect of insulin was investigated. Insulin significantly reversed the effect of Hcy on ferritin L and had a negligible effect on ferritin H at the same time (Fig. 4). Furthermore, insulin treatment restored Akt kinase phosphorylation and thus, the activity as observed in Hcy-treated cells.

**Fig. 5:**
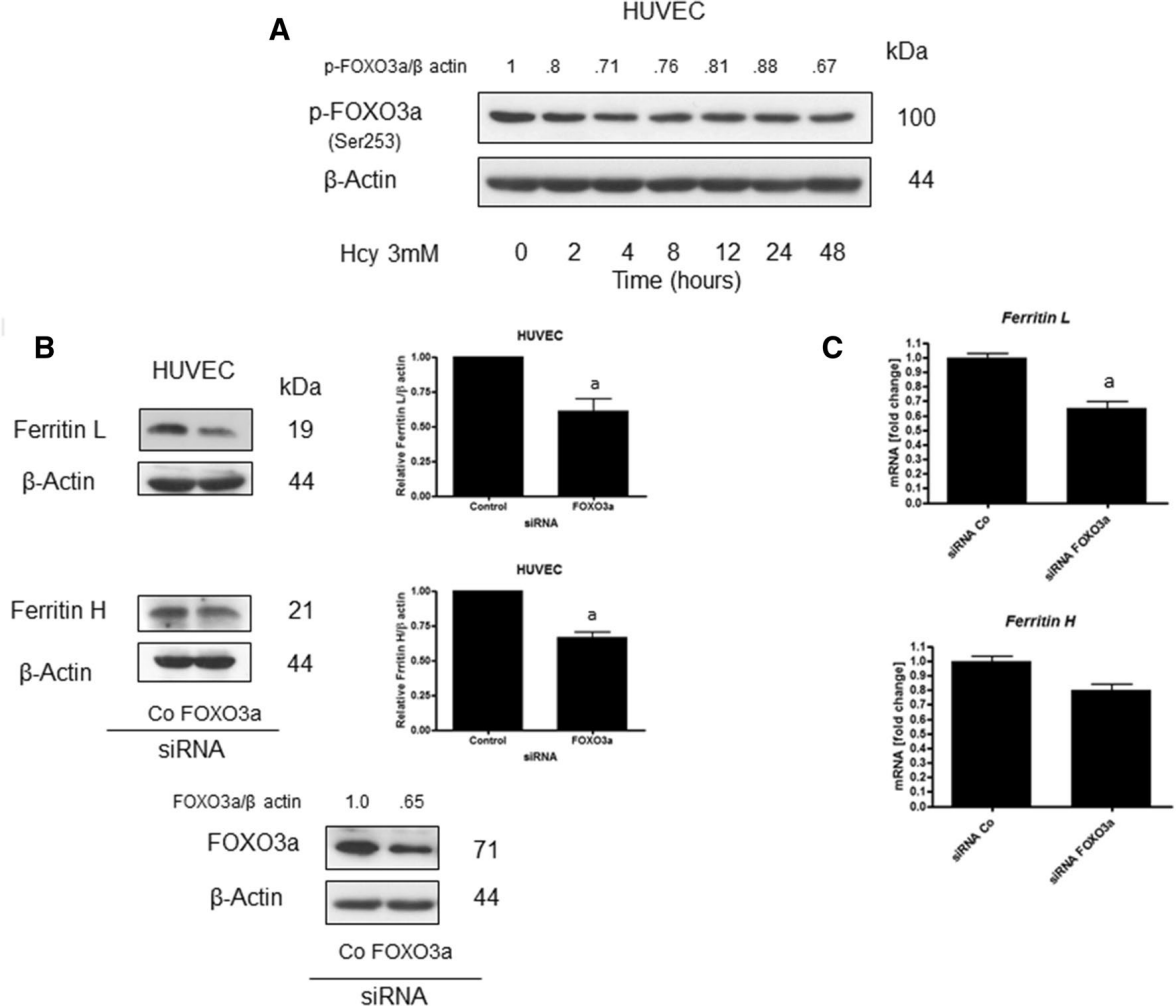
Role of FOXO3a in homocysteine-induced ferritin upregulation. **A** Immunoblotting for p-FOXO3a using lysate from HUVEC cells having been treated with 3 mM Hcy for the indicated time periods. The numbers on top of the immunoreactive bands represent a change in protein levels relative to control. **B** Immunoblotting for ferritin L, ferritin H and FOXO3a using lysate from HUVEC cells transfected with siRNA against FOXO3a. Blots were stripped and reprobed with anti-β-actin antibody to normalize for differences in protein loading. Bar graphs on the right panel of the figure present the mean ± SEM of three independent experiments relative to control where "a" represents statistical significance at *p* < 0.05 as compared to control (paired *t* test). **C** Ferritin L and ferritin H mRNA levels in HUVEC cell line transfected with siRNA against FOXO3a. mRNA levels were measured by qPCR. Data are shown as means ± SD of three independent experiments carried out in duplicate ("a" represents significance at *p* < 0.05, as compared with control siRNA transfected cells ( paired *t* test)

We speculated that FOXO3a, whose activity is under control of Akt, might regulate ferritin gene expression. Hcy treatment induced a drop in FOXO3a phosphorylation at a position which is responsible for its inactivation (Fig. 5A). To confirm a potential role of FOXO3a in ferritin upregulation, HUVEC cells were transfected with FOXO3a siRNA. Cells with downregulated FOXO3a manifested a significant decrease in ferritin H and L proteins level (Fig. 5B). In addition, mRNA levels of ferritin L were reduced in FOXO3a siRNA transfected cells (Fig. 5C). These data suggest that FOXO3a mediates ferritin upregulation in Hcy-treated cells.

### Hcy pre-treatment increased HUVEC sensitivity to hydrogen peroxide

**Fig. 6:**
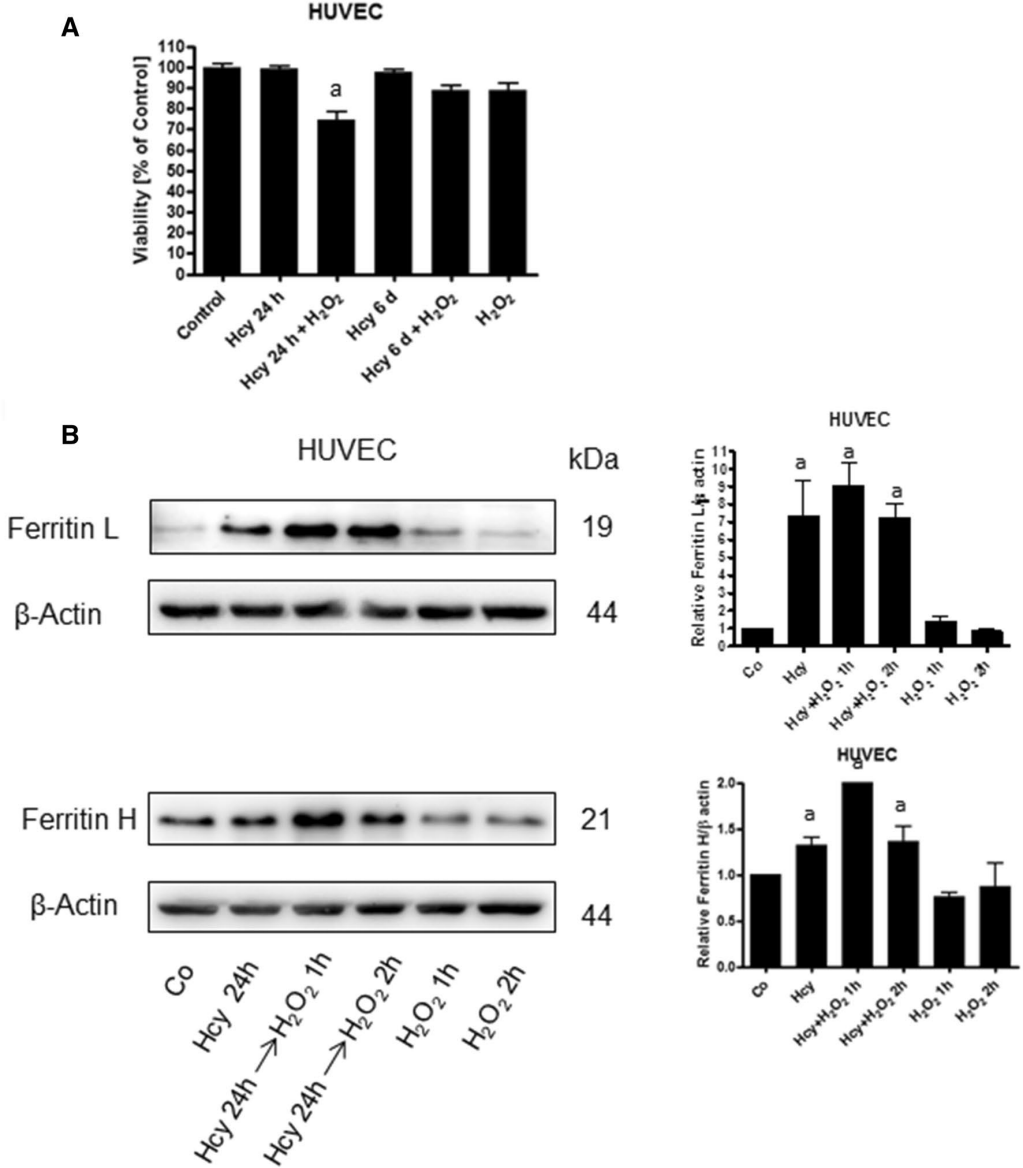
Homocysteine treatment decreases HUVEC cells' resistance to H_2_O_2_. **A** Survival of HUVEC cells following 24 h treatment with 300 μM H_2_O_2_ with or without 24 h pre-treatment with 3 mM Hcy. Cell viability was determined by trypan blue assay. Similar results were observed in at least three independent experiments. Data are presented as mean ± SE (*n* = 3); "a" represents statistical significance at *p* < 0.001 (one-way ANOVA followed by Tukey’s multiple comparison test). **B** Immunoblotting for ferritin L and ferritin H using lysates from HUVEC cells following 1 h or 2 h treatment with 300 μM H_2_O_2_ with or without 24 h pre-treatment with 3 mM Hcy. Blots were stripped and reprobed with anti-β-actin antibody to normalize for differences in protein loading. Bar graphs on the right panel of the figure present the mean ± SEM of three independent experiments relative to control where "a" represents statistical significance at *p* < 0.05 as compared to control (one-way ANOVA followed by Tukey’s multiple comparison test)

**Fig. 7:**
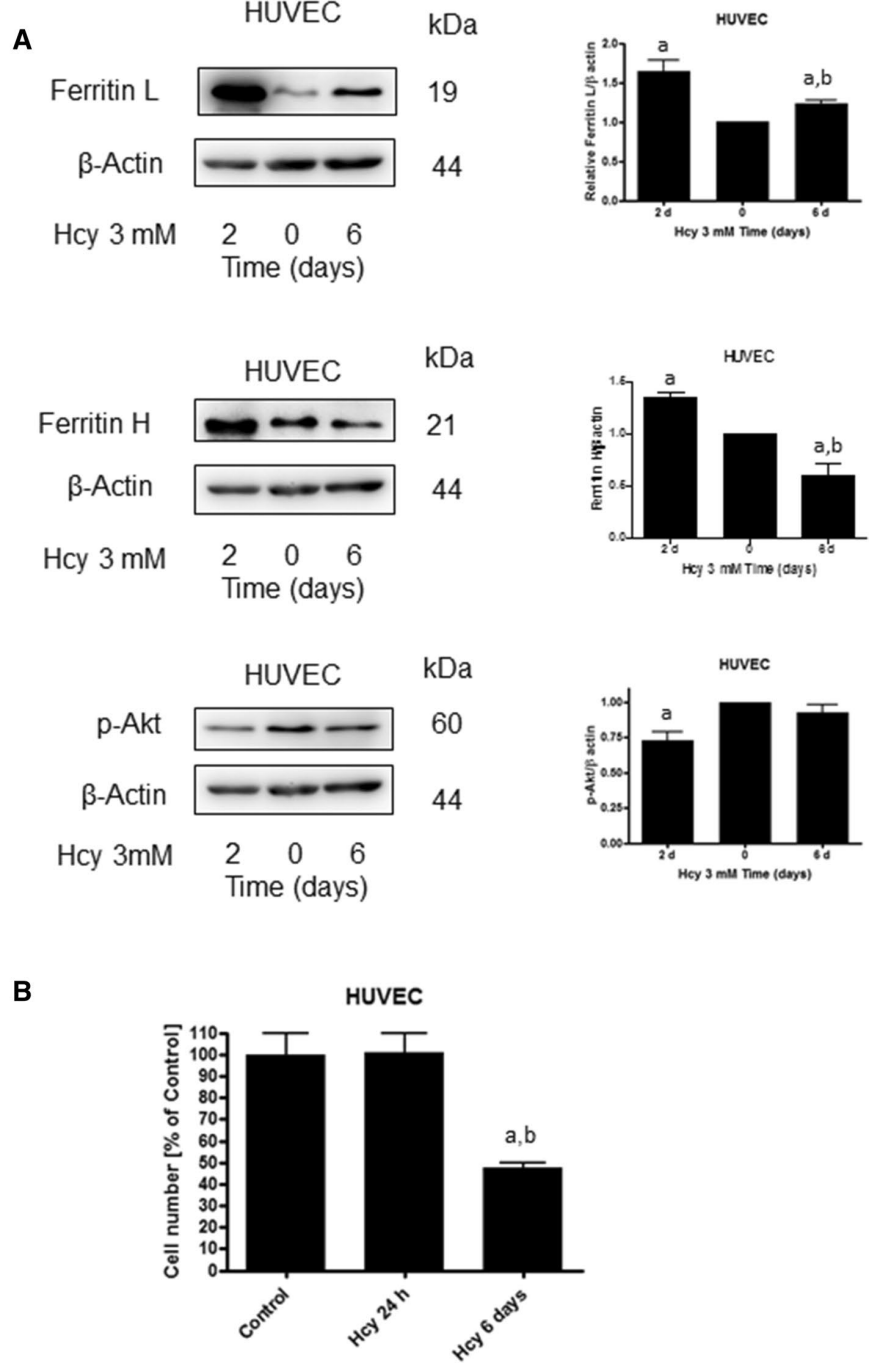
Effect of 6-day homocysteine treatment on p-Akt, ferritin and cell number. **A** Immunoblotting for ferritin L, ferritin H and p-Akt using lysate from HUVEC cells treated with 3 mM Hcy for the indicated time periods. Blots were stripped and reprobed with anti-β-actin antibody to normalize for differences in protein loading. Bar graphs on the right panel of the figure present the mean ± SEM of three independent experiments relative to control where "a" represents statistical significance at *p* < 0.01 as compared to control; "b" represents statistical significance at *p* < 0.001 as compared to 2 days (one-way ANOVA followed by Tukey’s multiple comparison test). **B** Number of HUVEC cells following 24 h or 6 days of treatment with 3 mM Hcy. Cell number was determined by trypan blue assay. Similar results were observed in three independent experiments. Data are presented as mean ± SE (*n* = 3); "a" represents statistical significance at *p* < 0.01 relative to control; "b" represents statistical significance at *p* < 0.01 relative to Hcy 24 h (one-way ANOVA test followed by Tukey’s multiple comparison test)

Ferritin is an iron storage protein which may protect the cell against iron toxicity but on the other hand, it can be a source of LIP. We determined the effect of Hcy pre-treatment on cellular sensitivity to hydrogen peroxide. We observed that the HUVEC cells pre-treated with Hcy for 24 h, whose ferritin level had increased relative to control, showed notably higher sensitivity to hydrogen peroxide (Fig. 6A). In HUVEC cells treated with Hcy and hydrogen peroxide, we demonstrated increased levels of ferritin H and L, similar to cells treated with Hcy alone (Fig. 6B). In addition to that, we observed that in HUVEC cells treated with Hcy for 6 days, ferritin L and H levels decreased and p-Akt level increased almost to control value (Fig. 7A). Moreover, the higher sensitivity of the cells to hydrogen peroxide vanished after 6 days of Hcy treatment (Fig. 6A). However, the number of HUVEC cells was significantly lower after exposition to Hcy for 6 days as compared to control or cells treated for 24 h with Hcy (Fig. 7B).

## Discussion

In this paper, we show for the first time that homocysteine treatment leads to changes in iron metabolism via impairment of Akt serine/threonine kinase signaling. We demonstrate that 3 mM Hcy-induced changes in iron metabolism in HUVEC cells are manifested by increased ferritin L and H protein levels, and increased sensitivity to hydrogen peroxide. Hcy concentration was selected on the basis of studies in which various concentrations of this compound were used (data not shown) and available literature data [17–19]. The observed changes in ferritin levels are at least partially mediated by decreased Akt activity and possible FOXO3a activation. Ferritin synthesis is controlled at both, transcriptional and translational level. An increased cellular iron import induces an adaptive response leading to the elevation of ferritin expression, while a decrease in iron level tends to inhibit ferritin biosynthesis. Based on the above, ferritin protein level has been considered as a good biomarker of the total amount of iron in a cell [20, 21]. An increased arterial ferritin accumulation was observed in pathologically changed arteries which is in accord with our data [22]. The mechanism responsible for ferritin accumulation is usually connected with an increase in the intracellular iron pool [23]. Deleterious changes in arteries were demonstrated to be triggered by different factors, like free fatty acids, pro-inflammatory cytokines, Hcy and others. Interestingly, most of these factors impaired signaling via insulin receptor leading to downregulation of PI3K/Akt with no stimulatory effect on the Shc/Pkc ERK signaling pathway [24]. In addition to that, decreased Akt activity, smaller NO synthesis and enhanced oxidative stress level were observed in hyperhomocysteinemic rats [4]. Moreover, there are reports demonstrating that changes in iron metabolism are associated with impairment of the PI3K/Akt signaling pathway. For example, ferritin H was shown to bind to CXCR receptor attenuating downstream signaling which in turn led to Akt inhibition [25, 26]. In this study, we observed that a drop in Akt phosphorylation, and thus its inactivation, takes place prior to the change in cellular ferritin proteins levels in HUVEC cells treated with Hcy whereas such effect was not observed in SH-SY5Y cells. Therefore, we speculated that a Hcy-induced decrease in Akt activity in HUVEC cells might trigger changes in iron metabolism rather than the other way around. Insulin was shown to reverse the Hcy-induced increase in ferritin L protein which partially confirmed our assumption about the role of Akt in the process. In addition, these data were confirmed in both, HUVEC (Fig. 3B) and SH-SY5Y cells (not shown) whose Akt1, Akt2 and Akt3 had been downregulated by siRNA. In those cells, a significant increase in ferritin proteins levels was observed indicating that impairment of signaling pathways downstream the insulin receptor-induced changes in iron metabolism. Akt belongs to a family of serine/threonine kinases which are activated by different kinds of growth factors, including insulin, IGF-1, CXCL12 and others. An impairment of Akt activity is observed in the endothelium from insulin-resistant subjects and in hyperhomocysteinemia [27]. PI3K/Akt metabolic pathways are involved in cell growth, migration and survival regulation. Among Akt substrates there is FOXO3a—a transcription factor characterized by a DNA binding domain called the “Forkhead box”. FOXO3a is known to regulate a wide range of cellular functions, including the cell cycle, apoptosis, atrophy, DNA repair and energy metabolism [28]. It is important to note that FOXO3a activation which could be a result of the Akt inactivation, also leads to an increase in both hydrogen peroxide scavenging and oxidative stress resistance [29]. Moreover, it has been demonstrated on *Caenorhabditis elegnas* model that DAF-16, modulates ferritin H gene expression [15]. Our data support this observation as in the cells with FOXO3a having been silenced by specific siRNA a decrease in ferritin L and H mRNA levels was observed.

Ferritin is an iron storage protein, and importantly, ferritin prevents iron ions from participation in redox reactions. However, ferritin can also be a source of LIP under stress condition. Thus, the observed increase in intracellular ferritin in cells treated with Hcy indicates that it is a result of the iron accumulation. We confirmed this assumption by showing that despite the surprisingly low toxicity of hydrogen peroxide itself cells pre-treated with Hcy were more sensitive to hydrogen peroxide as compared to non-pre-treated control, which is in accord with previous studies [29, 30]. The low toxicity of hydrogen peroxide is probably the result of using a complete medium which contains growth factors, serum, pyruvate. These are substances that have protective functions against oxidative stress-induced cell death. Besides, there are works on other research models, that show that despite the lack of morphological changes and specific damage in the cell, apoptosis processes are triggered in the right conditions, which are not initially visible [31]. Interestingly, the higher sensitivity of these cells was associated with a higher ferritin protein content. In addition, we observed that prolonged 6-day treatment with Hcy led to the restoration of Akt activity, ferritin protein content and sensitivity to hydrogen peroxide which supports the hypothesis of deleterious changes in cellular iron metabolism induced by Hcy. Changes in iron metabolism could initiate mechanisms which consequently lead to the inhibition of cell growth that was observed in our experimental set-up after 6-day incubation with Hcy.

It is possible that the observed increase in ferritin protein level is mediated by LIP. However, the data showed that the ferritin increase in cells treated with Hcy was also observed in the presence of iron chelator DFO. In general, ferritin is believed to play an antioxidative role in the cell, however, it can also be a source of redox-reactive iron ions which may enhance oxidative stress. For example, elevated expression of ferritin H in dopamine midbrain neurons of young animals resulted in iron accumulation, LIP expansion and neurodegeneration [32]. It was shown that ferritin undergoes proteasomal degradation and the process is mediated by stress-activated protein kinases and an adaptor protein p66Shc [13, 14]. In addition, some reducing agents can liberate iron from ferritin [33]. In fact, these and many other studies suggest that iron is not safely stored in ferritin [10, 34]. In our model, an initial decrease in ferritin protein level was observed at relatively early time points which evidently indicates ferritin degradation.

In conclusion, our study demonstrates for the first time that inactivation of Akt as observed in Hcy-treated cells leads to an increase in the cellular ferritin protein level which can be considered as an adverse response to the treatment. There is obviously much work to be done to elucidate a detailed mechanism of Hcy-iron interaction. Taking into account that many diseases are associated with impairment of Akt signaling pathway, one can speculate that changes in iron metabolism play a crucial role in their pathogenesis.
